# Genetic risk for schizophrenia is associated with altered visually-induced gamma band activity: evidence from a population sample stratified polygenic risk

**DOI:** 10.1038/s41398-021-01678-z

**Published:** 2021-11-16

**Authors:** S. I. Dimitriadis, G. Perry, S. F. Foley, K. E. Tansey, D. K. Jones, P. Holmans, S. Zammit, J. Hall, M. C. O’Donovan, M. J. Owen, K. D. Singh, D. E. Linden

**Affiliations:** 1 Neuroscience and Mental Health Research Institute, Cardiff University, Cardiff, CF24 4HQ UK; 2 Cardiff University Brain Research Imaging Centre (CUBRIC), School of Psychology, College of Biomedical and Life Sciences, Cardiff University, Cardiff, CF24 4HQ UK; 3 MRC Centre for Neuropsychiatric Genetics and Genomics, Division of Psychological Medicine and Clinical Neurosciences, Cardiff School of Medicine, Cardiff University, Cardiff, CF24 4HQ UK; 4 Neuroinformatics Group, School of Psychology, Cardiff University, Cardiff, CF24 4HQ UK; 5 Population Health Sciences, Bristol Medical School, University of Bristol, Bristol, BS8 1UD UK; 6 School for Mental Health and Neuroscience, Faculty of Health, Medicine and Life Sciences, Maastricht University, Maastricht, 406229 The Netherlands

**Keywords:** Predictive markers, Clinical genetics

## Abstract

Gamma oscillations (30–90 Hz) have been proposed as a signature of cortical visual information processing, particularly the balance between excitation and inhibition, and as a biomarker of neuropsychiatric diseases. Magnetoencephalography (MEG) provides highly reliable visual-induced gamma oscillation estimates, both at sensor and source level. Recent studies have reported a deficit of visual gamma activity in schizophrenia patients, in medication naive subjects, and high-risk clinical participants, but the genetic contribution to such a deficit has remained unresolved. Here, for the first time, we use a genetic risk score approach to assess the relationship between genetic risk for schizophrenia and visual gamma activity in a population-based sample drawn from a birth cohort. We compared visual gamma activity in a group (*N* = 104) with a high genetic risk profile score for schizophrenia (SCZ-PRS) to a group with low SCZ-PRS (*N* = 99). Source-reconstructed V1 activity was extracted using beamformer analysis applied to MEG recordings using individual MRI scans. No group differences were found in the induced gamma peak amplitude or peak frequency. However, a non-parametric statistical contrast of the response spectrum revealed more robust group differences in the amplitude of high-beta/gamma power across the frequency range, suggesting that overall spectral shape carries important biological information beyond the individual frequency peak. Our findings show that changes in gamma band activity correlate with liability to schizophrenia and suggest that the index changes to synaptic function and neuronal firing patterns that are of pathophysiological relevance rather than consequences of the disorder.

## Introduction

Higher neuronal synchronization within the gamma frequency range (30–90 Hz) has been linked to several core cognitive processes, including memory performance [1], attention [2], object recognition [3], and motor control [4]. In primates, high-contrast grating stimuli generate induced (i.e., not phase-locked to stimulus onset) gamma oscillations in local field potential (LFP) recordings from the primary visual cortex (V1) [5, 6], and high contrast image patterns increase the coherence between neuronal populations in V1 [7, 8].

Comparable effects can be captured non-invasively in humans using magnetoencephalography (MEG) [9–12], and there is evidence that the sustained narrow-band visual gamma response is generated in V1 [13, 14]. Properties of visually induced gamma oscillations are related to perceptually important properties of visual stimuli—such as contrast [13, 15], orientation [16, 17], size [18], spatial frequency [9, 19], and temporal frequency [12].

There is well-established evidence that physiological and behavioral deficits in schizophrenia are associated with impaired perceptual processing; for example, abnormalities of the visual system have been demonstrated in patients with schizophrenia and their unaffected relatives using non-invasive physiological methods [20–24], post-mortem anatomy [25], and psychophysics [26, 27]. In addition, seral EEG and MEG studies have shown an association between altered high-frequency oscillations and visual deficits in patients with schizophrenia [22, 23, 28, 29]. Thus, dysfunction in high-order cortical areas that support more complex cognitive functions could be explained by perceptual deficits of bottom-up processing of incoming stimuli [30].

However, findings from MEG studies comparing patients and controls have not been consistent regarding the direction of effects and the same frequency bands implicated. Limitations of patient studies include the biological heterogeneity of unstratified patient groups, potential secondary effects of the illness, and potential medication confounds in many studies. Studies in genetically identified risk groups can overcome these limitations of reverse causation. In recent years, genome-wide association studies have identified a large number of shared alleles that are associated with schizophrenia [31, 32]. While individually, these explain only a small proportion of risk for the disease, collectively, they contribute around a third to a half of the total genetic risk for schizophrenia [33]. The identified risk alleles make it possible to stratify individuals according to a schizophrenia risk profile (SCZ-PRS) that indexes liability to the disorder based on their burden of risk alleles. The relationship between genetic liability, as defined by the PRS, and putative biomarkers for schizophrenia can then be investigated even in individuals without schizophrenia who are free of disorder—and treatment-related confounds [34].

There is a clear case for investigating visual gamma activity as one potential biomarker: not only does it have a hypothesized relationship with schizophrenia, as outlined above, but spectral properties of the visual gamma response have also been shown to be highly heritable [35]. Furthermore, genes coding for components of GABAergic and glutamatergic signaling processes, which maintain the balance between excitatory and inhibitory activity reflected in gamma activity, are enriched for risk variants for schizophrenia [36]. Thus, in the present study, we used individuals stratified by liability to schizophrenia to explore, for the first time, whether visually induced gamma activity differs between individuals with extreme high and low SCZ-PRS.

## Materials and methods

### Participants

Participants were recruited by the Avon Longitudinal Study of Parents and Children (ALSPAC) birth cohort by polygenic risk for schizophrenia. This broader cohort consisted of 14,062 children born to women residing in the Avon Health Authority area, from April 1, 1991, to December 31, 1992 (http://www.bristol.ac.uk/alspac/; available at http://www.bristol.ac.uk/alspac/researchers/access/). Pregnant women in Avon, the UK, with expected delivery dates April 1, 1991, to December 31, 1992, were invited to participate in the study. The initial number of pregnancies enrolled is 14,541 (for these, at least one questionnaire has been returned, or a “Children in Focus” clinic had been attended by 19/07/99). Of these initial pregnancies, there were 14,676 fetuses, resulting in 14,062 live births and 13,988 children who were alive at one year of age [37–39]. Polygenic risk scores for schizophrenia (SCZ-PRS) have been estimated for *N* = 8169 children following a normal distribution. For a recent multi-modal neuroimaging study, we attempted to recruit 200 subjects from the extremes of this distribution to create two groups of 100 subjects with high and low SCZ-PRS, matched on sex. The Central Bristol Research Ethics Committee approved the study (13/S.W./0170), and the local research ethics committees (listed at http://www.bristol.ac.uk/alspac/researchers/research-ethics/).

All participants were recruited from ALSPAC based on their PRS for schizophrenia. The ALSPAC team sent out 1241 invitations (470 to the low and 771 to the high SCZ-PRS group). Individuals were excluded if they were receiving any psychotropic medication. We ultimately assessed 203 individuals—99 (52 female, 47 male) individuals with low SCZ-PRS and 104 individuals (52 female, 52 male) with high SCZ-PRS age-matched from either tail of the SCZ-PRS distribution from a large genotyped population (see Fig. 1 in Lancaster et al., 2019). The mean group z-PRS was above 1.5 (1.42 higher than the mean PRS for the high, 1.71 lower than the mean PRS for the low group). The two groups did not differ on age (low SCH-PRS 22 years and one month ± 10 months, high SCH-PRS 22 years and 2 months ± 8 months with a *p*-value = 0.33, Wilcoxon rank-sum test). Psychotic experiences (hallucinations, delusions, or experiences of thought interference) were assessed using the semi-structured Psychosis-Like Symptom Interview at 18 years of age. We observed for *n* = 172 subjects a 1.00 to 1.20 [lower 95%, higher 95%] for psychotic experiences (see Table 1 in Lancaster et al., 2019). Ethical approval for the study was obtained from the ALSPAC Ethics and Law Committee and the Local Research Ethics Committees. Informed consent for the use of data collected via questionnaires and clinics was obtained from participants following the recommendations of the ALSPAC Ethics and Law Committee at the time. For further details about this cohort and the multi-modal imaging protocol (see ref. [34]).

**Fig. 1:**
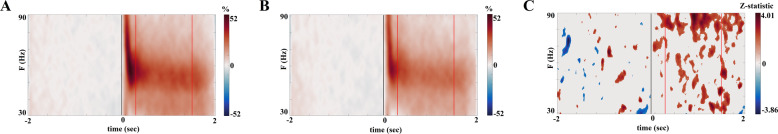
Group-averaged time-frequency plots of visual-stimulus induced activity. **A** Low-risk, **B** high-risk groups, and **C** Zstatistical mapping of group differences across time-frequency dimensions. Color refers to the Z-statistic accompanied Wilcoxon Signed Rank Sum Test. Positive *Z*-statistic values (red color) refer to strong evidence that visual-stimulus induced activity is higher for the low SCZ-PRS group than high SCH-PRS. Negative Z-statistic values (blue color) refer to strong evidence that visual-induced activity is higher for high SCZ-PRS than for the low SCZ-PRS group. The *x*-axis denotes the pre and post-stimulus time period (–2 to 2 s), while the y-axis refers to the frequency response (Hz). The color scale represents amplitude as % change relative to the baseline period (–1.5 to 0 s). Thus, the black line orients the starting point of the active task (0) while the two red vertical lines denote the time limits of transient stimulation period (0.3 s) and sustained period (1.5 s) used for the spectral frequency analysis of visual-stimulus induced activity shown in Figs. 2 and 3, respectively.

**Table 1:** OR and *β* coefficients (±95% confidence intervals) for psychotic experiences and WISC-III IQ measures by SCZ-PRS group (higher OR/coefficients reflect an association with the high SCZ-PRS group).

Phenotype	Estimate	Lower 0.95%	Upper 0.95%	*p*
Psychotic experiences	1.100^a^	1.00660	1.20283	0.039
WISC-III (verbal)	0.217^b^	−4.48233	4.91643	0.927
WISC-III (performance)	1.944^b^	−2.83438	6.72296	0.423
WISC-III (total)	1.606^b^	−2.77053	5.98341	0.470

^a^Odds ratio (OR).

^b^*β* coefficients.

Study data were collected and managed using REDCap (Research Electronic Data Capture) electronic data capture tools hosted at CUBRIC Neuroimaging Centre [40, 41]. REDCap is a secure, web-based software platform designed to support data capture for research studies, providing (1) an intuitive interface for validated data capture; (2) audit trails for tracking data manipulation and export procedures; (3) automated export procedures for seamless data downloads to standard statistical packages; and (4) procedures for data integration and interoperability with external sources.

Please note that the study website contains details of all the available data through a fully searchable data dictionary and variable search tool and reference the following webpage: http://www.bristol.ac.uk/alspac/researchers/our-data/.

### Stimulus and procedure

During MEG data acquisition, participants visually presented grating stimuli. The stimuli were stationary, vertically oriented, luminance-defined, square-wave gratings with three cycles/degree spatial frequency. Each stimulus was masked by a square window measuring 8° × 8° and presented centrally at maximum contrast on a mean luminance (26.5 cd/m^2^) gray background. The stimuli were generated by MATLAB (The Mathworks, Inc., Natick, MA) Psychophysics Toolbox extensions [42–44] and presented on a Mitsubishi Diamond Pro 2070 monitor (1024 × 768 pixel resolution, 100 Hz refresh rate).

Each subject performed 100 trials consisting of a 2000 ms baseline period, followed by the presentation of the stimulus for a random duration between 1500 and 2000 ms, followed by a 1000 ms response period, a total trial time of 4500–5000 ms. A red square (~0.2° in width) was present continuously during each trial, and participants were instructed to maintain fixation on the square throughout. In addition, to encourage participants to maintain attention to the stimuli, they were instructed to respond to stimulus offset from the screen by pressing a single button with the index finger of their right hand as rapidly as possible.

### MEG/MRI data acquisition and analysis

GP, SID, and SF who conducted MEG/MRI experiments were blinded to the group allocation. Whole-head M.E.G. recordings were made using a 275-channel CTF radial gradiometer system sampled at 1200 Hz. An additional 29 reference channels were recorded for noise cancellation purposes, and the primary sensors were analyzed as synthetic third-order gradiometers [45]. Unfortunately, three of the 275 channels were turned off due to excessive sensor noise.

We achieved MRI/MEG co-registration by replacing fiduciary markers at fixed distances from three anatomical landmarks (nasion and pre-auricular) identifiable in the subjects’ anatomical MRIs.

Data were acquired as a single continuous acquisition and segmented post-hoc into 4 s epochs beginning at 2 s before stimulus onset, each epoch corresponding to an experimental trial. Artifact rejection was performed offline by manually inspecting the data and discarding epochs with excessive muscle or head-movement-related artifacts. No more than 10 trials out of 100 were excluded from any individual dataset in this way. A number of trials after artifact rejection did not significantly differ between low and high SCZ-PRS groups (Wilcoxon Rank-Sum Test, *p* = 0.96).

Participants also underwent magnetic resonance (MR) data acquisition on a 3 T GE scanner with an 8-channel receive-only head RF coil. For MEG source localization purposes, we obtained a 3D FSPGR scan with 1 mm isotropic voxel resolution and used this to derive a multiple local-spheres forward model [46] (Huang et al., 1999) by fitting spheres to the brain surface extracted by FSL’s Brain Extraction Tool [47] (Smith, 2002). Based on this forward model, the synthetic aperture magnetometry (SAM) beamformer [48] was applied to create *t*-statistical images of the difference in source power between visual stimulation (0–1.5 s) and baseline (−1.5 to 0 s) across all trials in each participant. Beamformer weights were calculated using the covariance matrix estimated from all epoched data after bandpass filtering within the broadband gamma range (30–90 Hz). Estimates of the three-dimensional distribution of source power were derived for each participant’s whole head at 4 mm isotropic voxel resolution.

Manual inspection of the SAM images demonstrated that most participants had at least one positive peak activity in the occipital cortex. Therefore, the location of this peak (or the peak with the largest *t*-statistical value if multiple peaks were present) was extracted and used as the source location for a ‘virtual sensor’ analysis. Because the MEG beamformer images do not have the spatial resolution to measure separate responses for the different visual areas (e.g., V1, V2), our approach is to extract virtual sensor time series to represent ‘bulk’ gamma activity within the early visual cortex. We define this location as the voxel in the beamformer image with the largest positive *t*-statistical differences between stimulus and a baseline maximize SNR in the virtual sensor time series.

Virtual sensor time series were generated for each condition per participant using the SAM beamformer method a second time for this single obtained location per individual. Unfortunately, for 11 participants (7 in the low and 4 in the high SCZ-PRS groups), we were not able to source-localize the gamma response, either because we were unable to acquire MR scans for those individuals or because their SAM images contained no positive peak in the occipital cortex (indicating that their visual gamma response was too weak to be measured by MEG). Therefore, these individuals were excluded from further analysis.

It has previously been shown that in a minority of individuals, the amplitude of the visual gamma response is too weak to allow reliable identification of the response frequency. Extracting these individuals from the analysis can improve the detection of between-condition differences in the gamma response [49]. Therefore, to quality control the data, we adopted a bootstrapping procedure of 10,000 iterations where trials were resampled with replacement, and for each resampling of the data, the peak gamma frequency was estimated using the methods described below “Time–frequency analysis of virtual time series”. We then used the distribution of peak frequencies as a quality control procedure for evaluating the reliability of the estimated peak frequencies. As a quality control criterion, we stipulated that a frequency range of <±2 Hz should encapsulate at least 50% of the bootstrapped frequencies around the distribution model. We also excluded subjects that passed this first criterion by estimating a *z*-score of the gamma frequency peak amplitude and excluding those with *z*-score <2.

This quality control process led to the exclusion of 25 subjects with low SCZ-PRS and 34 with high SCZ-PRS, meaning that the final number of subjects per group was 67 for the low SCZ-PRS and 66 for the high SCZ-PRS.

#### Time–frequency analysis of virtual time series

The main objective of time–frequency analysis of virtual ‘visual’ time series is to report group differences in time–frequency and spectral domains. Thus, both types of analysis provided below are exploratory across time, frequency, and spectrum dimensions.

For each participant, time–frequency analysis of the single-trial virtual sensor time series was then performed by means of bandpass filtering with an 8 Hz bandwidth at center frequencies between 30 and 90 Hz and calculating the Hilbert envelope. The resulting spectrograms were calculated separately for each trial and then averaged to reveal induced responses. The results from time–frequency analysis are shown in the section “Time–frequency analysis of virtual time sensor series” and Fig. 1. The magnitude for each time–frequency data point was calculated as the percentage change in amplitude relative to the average amplitude at that frequency in the baseline period (−1.5 to 0 s) (Fig. 1). To calculate the induced response (which provides information about the power of non-phase locked activity), the relative change in power was estimated separately for every trial and then averaged (Fig. 1A, B). Wilcoxon signed-rank test statistic was used to demonstrate the effect-size of group differences in the percentage change from baseline across the spectrum and temporal dimensions (Fig. 1C).

In a primary analysis, for each participant, gamma peak frequency within 30–90 Hz, amplitude and latency of the maximum percent change of the visual stimulation compared to baseline (−1.5 to 0 s) were extracted from the time–frequency data within the gamma spike time period (0–0.3 s). Statistical contrasts of these measures were then performed between the two groups (see the section “Group differences in gamma peak amplitude, latency, and frequency in the virtual sensor space”). Thus, our primary hypothesis pertained to a group difference in peak frequency, with differences in amplitude and latency as secondary analyses.Wilcoxon signed-rank test statistic was used to demonstrate the effect-size of group differences in gamma peak frequency within 30-90 Hz, amplitude and latency of the maximum percent change of the visual stimulation compared to baseline (−1.5 to 0 s).

In an exploratory analysis, we estimated the spectral power of trial-by-trial responses in the frequency domain from the active period as percentage change to the baseline. The whole analysis was repeated separately for two active periods: the gamma spike time period (0–0.3 s) and the sustained period (0.3–1.5 s) as in our previous studies [18, 49]. The initial evoked gamma onset “spike” is visible at around 130–250 ms [12] after the onset of the stimulus, while simple contrast pattern stimuli induce sustained narrow-band gamma oscillations generated in the visual cortex [18, 29]. The rationale for analyzing these time periods separately is that multiple prior studies have shown distinct responses in these time periods [29, 50, 51] and likely have different generative mechanisms. The gamma spike is likely related to the visual input through the retina–geniculostriate pathway, while the sustained period involves cortical feedback mechanisms [29, 52, 53].

**Fig. 2:**
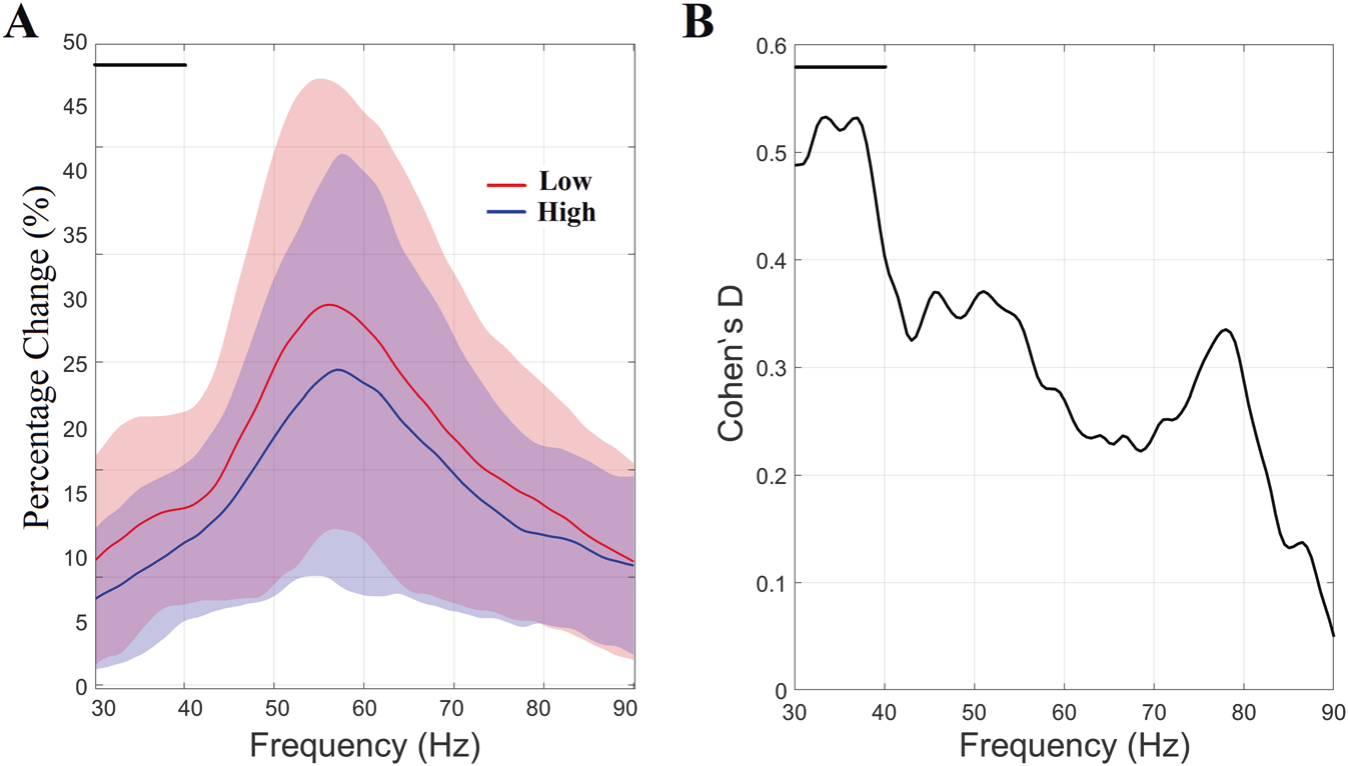
Percentage change (%) of relative gamma spectrum related to spike activity in the transient period (0 to 0.3 s). **A** Percentage change (%) of gamma spike activity in the transient period (0 to 0.3 s), compared to the baseline (–1.5 to 0 s), averaged within each group (shaded areas represent +/– 1 SD). Horizontal black lines indicate the gamma range where a medium effect of group difference was detected within 30–40 Hz. **B** Cohen’s D statistic estimated per frequency bin.

**Fig. 3:**
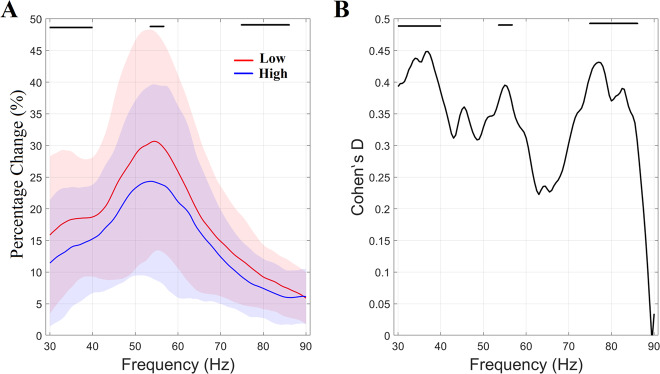
Percentage change (%) of relative gamma spectrum related to the active (sustained) period (0.3 to 1.5 s). **A** Percentage change (%) of sustained activity in the active (sustained) period (0.3 to 1.5 s) compared to the baseline (–1.5 to 0 s) averaged within each group (shaded areas represent +/– 1 SD). Horizontal black lines indicate the three gamma ranges where small to medium group differences were detected within (a) 30–40 Hz, (b) 54–56.5 Hz, and (c) 75–86 Hz. **B** Cohen’s D statistic estimated per frequency bin.

We averaged time–frequency data across time within each of the two periods (0–0.3 and 0.3–1.5 s) and the baseline period (−1.5 to 0 s). Then, the estimated characteristic spectrum value in the active time window was represented as a percentage change from baseline. This procedure was repeated for every frequency bin within 30–90 Hz. The results from the spectral analysis estimated within the active period are shown in the section “Time-averaged spectral analysis of the virtual sensor time series” and Fig. 2 (gamma spike) and Fig. 3 (sustained gamma). Cohen’s *d* statistic was used to demonstrate the effect-size of group differences in the percentage change from baseline in both time periods.

## Results

### Psychopathology and cognition

We reported associations between psychotic experiences and WISC measures with SCZ-PRS from available individuals. We observed a nominal association between an increased incidence of psychotic experiences (*n* = 172) and high SCZ-PRS group allocation (psychotic experiences in low [*N* = 5; 5.75%] and high [*N* = 12; 17.65%] SCZ-PRS, *P* = 0.039). We observed no association between SCZ-PRS and any IQ. dimension (*n* = 183) (Table 1).

### Group differences in gamma peak amplitude, latency, and frequency in the virtual sensor space

We tested for group differences over the peak visual gamma response (amplitude, frequency, and onset latency). Group-mean gamma peak frequencies were 52.6 Hz (±8.9) for the group with low SCZ-PRS and 52.5 (±8.3) for the group with high SCZ-PRS (*p* = 0.78). Group-mean gamma amplitudes were 176.57 nAM (±79.53) for the group with low SCH-RPS and 187.17 nAM (±76.99) for the group with high SCH-RPS (*p* = 0.91). Group-mean gamma onset latencies were 0.12 s (±0.02) for the group with low SCZ-PRS and 0.11 s (±0.03) for the group with high SCZ-PRS (*p* = 0.89). We used the Wilcoxon Signed Rank Sum Test to estimate the p-values.

### Time–frequency analysis of virtual time sensor series

Average spectrograms of the visual-stimulus-induced response within each group revealed the patterns illustrated in Fig. 1A, B. First, we compared individual time–frequency differences between the two groups on a fine-grained resolution across both time and frequency dimensions via Wilcoxon Signed rank-sum Test corrected with false discovery rate (*q* = 0.01) for multiple comparisons (121 frequency bins × 720 time points). Then, applying an absolute threshold at the resulting *p*-values (*p*-value < 0.05), we illustrated the related *Z* statistical mapping across time–frequency dimensions revealing Spatio-temporal groups of time-frequency points (121 frequency points × 4800 time points) where the two groups differ (Fig. 1C). Positive *Z*-statistic values (red color) refer to strong evidence where visual-stimulus-induced activity is higher for the low SCZ-PRS group than high SCH-PRS for many pairs of subjects. Conversely, negative *Z*-statistic values (blue color) refer to strong evidence that visual-induced activity is higher for high SCZ-PRS than for the low SCZ-PRS group.

### Time-averaged spectral analysis of the virtual sensor time series

Group-averaged relative spectrum profiles, averaged over the first transient stimulation time period [0–0.3 s], revealed strong evidence of a difference, as demonstrated in Fig. 2A. A Wilcoxon Signed rank-sum Test corrected with false discovery rate (*q* = 0.01) for multiple comparisons (across 121 frequency bins) revealed a strong trend of increment in relative power spectrum for the low, compared to high, SCH-PRS group within the frequency interval 30–40 Hz. Cohen’s *D* effect-size measures, shown in Fig. 2B, demonstrated a medium effect size of 0.5 for this gamma range.

Group-averaged relative spectrum profiles, averaged over the second, sustained, stimulation time period [0.3–1.5 s], also revealed a strong effect demonstrated in Fig. 3A. A Wilcoxon Signed rank-sum Test corrected with false discovery rate (*q* = 0.01) for multiple comparisons (across 121 frequency bins) revealed a substantial reduction in the relative spectrum for the high SCH-PRS group within three frequency intervals: (a) 30–40 Hz, (b) 54–56.5 Hz and (c) 75–86 Hz. Cohen’s *D* effect-size measures, shown in Fig. 3B, demonstrated small to medium effect sizes (0.38–0.43) for these gamma ranges.

## Discussion

For the first time, we investigated whether the visually induced gamma response differs between general population individuals at extreme ends of the distribution of a polygenic risk score for schizophrenia (SCZ-PRS). In our primary analysis, peak amplitude, frequency and latency of the sustained gamma response did not show any substantial difference between groups, and thus we could not reject the null hypothesis of no group differences in parameters of the peak response. In the exploratory analysis, group-averaged visual stimulus gamma spike activity in the frequency interval 30–40 Hz, expressed as a percentage change relative to the baseline, was lower in the high SCZ-PRS group compared to the low SCZ-PRS group (Fig. 2), with a medium effect size. Additionally, group-averaged visual-stimulus-induced gamma (sustained) activity expressed as a percentage change relative to the baseline revealed a reduction in three frequency gamma frequency intervals (30–40, 54–56.5, and 75–86 Hz) for the high SCZ-PRS group, compared to the low SCZ-PRS group (Fig. 3), with a small to medium effect size. Our findings thus add to the evidence supporting the use of visual gamma (VG) as a biomarker that can be sensitive to the liability of schizophrenia and particularly the mechanisms of genetic risk. There is evidence that the gamma spike in the first 300 ms (transient period) is driven by the stimulus and reflects incoming firing rates of neurons in V1 via the M pathway from the retina via the lateral geniculate nucleus (LGN) [54]. Computational modeling of contrast and orientation tuning has revealed the link of visual abnormalities to the continuum progress of schizophrenia starting from the first psychotic episode to its chronic stage [53]. The primary visual cortex exhibits two distinct gamma bands: a broadband gamma activity characterized by transient increases and stimulus onset and a narrow-band gamma activity that shows a more sustained temporal profile. A recent invasive study in humans revealed the distinct role of narrow and broadband gamma responses, supporting our analysis of gamma sub-bands [55]. This narrow-band sustained gamma activity appears to be related to inhibition within the visual system [56] and hence may be a signature of inhibitory gain control [29]. Reduced sustained gamma is, therefore, an indicator of reduced inhibitory control. The broadband gamma rhythm is likely related to the spiking firing rate of pyramidal cells, while the narrow-band gamma originates from pyramidal local field postsynaptic potentials [29, 57].

Our findings are consistent with studies in patients with schizophrenia that highlight aberrant neural responses in visual areas due to either deficit in sensory perception and/or the propagation of feedforward information [58, 59]. A previous MEG study revealed a decrease of visually induced gamma activity in schizophrenia patients at frequencies >60 Hz [23]. In the present study, high SCH-PRS demonstrated a small to medium effect of lower relative power changes in the active period compared to the baseline compared to the low SCH-PRS within three beta/gamma ranges (30–40, 54–56.5, and 75–86 Hz) (Fig. 3). This finding suggests that oscillatory activity is a sensitive marker of altered brain function in people with a high genetic risk of schizophrenia.

Previous research has found that the frequency, amplitude, and spectral profile of visual gamma oscillations measured with MEG recordings [11] are consistent within subjects over several weeks. More recently, gamma frequency, amplitude, and bandwidth have been demonstrated to have high test–retest reliability at sensor and source levels using MEG [60]. This reliability of the gamma response within-subjects may partially explain why we can find such consistent differences between groups.

Importantly, visual gamma activity is sensitive to the properties of visual stimulation [61] and is modulated by attention [62]. There is also evidence that it may involve the feedforward propagation of sensory information through the visual hierarchy [63].

It has been shown that disruptions of gamma oscillations in cortical areas are partly driven by disruption of GABA transmission [64]. There is evidence from modeling studies that changes in the kinetics of GABAergic response will affect both the frequency and the power of gamma oscillations [65, 66]. Animal modeling studies have demonstrated that a reduction in the glutamatergic input to interneurons can also strongly affect the power of gamma oscillations [67]. However, interneurons and principal cells also contain many ion channels that support their ability to sustain this fast oscillatory activity. Integrating the findings mentioned above, we can infer that gamma activity depends upon the balance between excitation and inhibition mediated by GABAergic interneurons and NMDA/AMPA-receptors in local neural circuits that control the duration and strength of the oscillations [67–69].

In patients with schizophrenia, there is evidence for alteration of the parameters of excitatory/inhibitory networks [70], including reductions in the mRNA of GAD67, an enzyme responsible for synthesizing a high proportion of GABA [71]. Furthermore, a reduction in GABA levels measured with magnetic resonance spectroscopy (MRS) is correlated with impairment in an orientation-specific surround suppression task in schizophrenia patients [72]. The reduction of the gamma frequency response of the visual cortex in the group with high SCH-PRS in our data may thus be linked to impairments in GABAergic neurotransmission [73, 74]. Alteration in GABAergic neurotransmission is not the only potential mechanism for altered high-frequency oscillations, however. An increase in spontaneous gamma activity has been linked to NMDA-R antagonists like ketamine [75]. Acute NMDA-R increases the amplitude and frequency of task-based MEG gamma oscillations in visual cortices in first-episode and chronic schizophrenia patients [76]. The reduction of gamma activity in the high SCZ-PRS observed in our study could be owed to the inhibition of NMDA-R receptors in local visual circuits. A mechanistic link between NMDA-R dysfunction, a putative pathomechanism of schizophrenia, and altered gamma activity is provided by the prominent role of genes coding for synaptic proteins in both standard and rare risk variants for the disease [36, 77–80] including the most recent and most extensive GWAS (Schizophrenia Working Group of the Psychiatric Genomics Consortium et al., 2020) and exome sequencing studies [81].

Previous MEG visual gamma studies have also suggested that gamma-band responses from the primary visual cortex are shaped by GABA_A_receptor-mediated inhibitory neurotransmission. Dysregulation of GABA_A_ receptors has been related to psychiatric disorders, including schizophrenia and depression [82]. Individual gamma oscillatory frequency has been positively correlated with resting GABA concentration levels in the visual cortex [83]. That study linked neuroimaging metrics like gamma frequency with the excitation/inhibition balance in the individual’s visual cortex [83]. Another MEG visual gamma-band study revealed a GABAergic synaptic disconnection in schizophrenic patients, a supportive mechanism of excitation/inhibition balance [29]. Finally, a previous MEG study reported an association between impaired power, connectivity, and variability of high-frequency oscillations in clinically high-risk subjects with impaired functioning and cognitive deficits [84].

Any effect of PRS on neural measures in our population study could have its origin also from non-transmitted alleles of the parental genotypes, inducing so-called “dynastic effects” [85]. Alterations of brain function could reflect environmental exposures that are correlated with parental genotypes. For example, effects of “genetic nurture,” produced by non-transmitted alleles, on offspring outcome were shown for educational attainment [86].

In summary, we have used common variant polygenic risk scoring to stratify a population for schizophrenia risk. By testing individuals at extreme ends of the risk distribution, we identified a distinct profile of the visual gamma response that links altered oscillatory activities observed in patient samples to individuals at genetic risk of the disorder. Because we recruited from a population cohort, we can have confidence that this effect is not due to drug- or disease-related confounds but instead is a candidate marker for neural effects of the genetic risk for schizophrenia. We suggest that further replication is needed to validate our findings.
